# Supplementary material for: Anxiety levels among school-going adolescents in peri-urban areas of Karachi, Pakistan
Source: PLoS One. 2023 Oct 19;18(10):e0289967. doi: 10.1371/journal.pone.0289967 (PMC10586665; doi:10.1371/journal.pone.0289967)
Supplement: Attachment — Submitted filename: plos one review.docx [file pone.0289967.s001.docx]

**Anxiety levels among school-going adolescents in peri-urban areas of Karachi, Pakistan**

**Abstract**

1. Please include rationale of the study and what this study adds to the exiting evidence.
2. Methods: “self-generated”???
3. Please revisit the methods section and make it more specific
4. Please rewrite the abstract

**What this study adds:** please rewrite the entire section to highlight the key findings and strengths of the study. Please revisit this text mentioned in the manuscript. *“Pakistan is facing a serious challenge in ensuring the enrolment and retention of children and adolescents at schools [1]. There is a dire need to ensure such an education system in the country.”*

**Introduction:** please rewrite and rationale for the study needs to be inbuilt within the background section

**Objectives:** The authors have mentioned 8 objectives in the manuscript. Please mention a comprehensive objective and rewrite.

Methods: the authors mention “systemic random sample” in sample size section (which is refereed as data size in the manuscript). Please revise to systematic.

Please clarify self-made form.

How was the questionnaire developed and validated?

Please mention about calibration of instruments as well.

Findings need to be rewritten and presented in a comprehensive manner.

Please rewrite conclusion and recommendations section.

The authors need to edit the entire manuscript for language and grammar.
