# Supplementary material for: Anxiety levels among school-going adolescents in peri-urban areas of Karachi, Pakistan
Source: PLoS One. 2023 Oct 19;18(10):e0289967. doi: 10.1371/journal.pone.0289967 (PMC10586665; doi:10.1371/journal.pone.0289967)
Supplement: Attachment — Submitted filename: REVIEWER 2.docx [file pone.0289967.s002.docx]

|  | **RESPONSE TO REVIEWER & EDITOR** |  |
| --- | --- | --- |
| 1 | 1. Please include rationale of the study and what this study adds to the exiting evidence | Thanks reviewer, we have added in revised manuscript |
|  | 2. Methods: “self-generated”??? | we have replaced the present from the following statement  ***Self-designed structured pre-tested questionnaires***. |
|  | 3. Please revisit the methods section and make it more specific | We have revised the method section and it will shown in track changes file |
|  | 4. Please rewrite the abstract | The abstract we have changed from previous to submitted one and made it more clear and specific to the study point of view |
|  | 5. What this study adds: please rewrite the entire section to highlight the key findings and strengths of the study. Please revisit this text mentioned in the manuscript. “Pakistan is facing a serious challenge in ensuring the enrolment and retention of children and adolescents at schools [1]. There is a dire need to ensure such an education system in the country.” | We have revised the rationale of the study |
|  | 6. Introduction: please rewrite and rationale for the study needs to be inbuilt within the background section | We have revised the introduction part and cite with supporting literature. |
|  | 7. Objectives: The authors have mentioned 8 objectives in the manuscript. Please mention a comprehensive objective and rewrite. | We have concise from 8 to 4 objectives |
|  | 8. Methods: the authors mention “systemic random sample” in sample size section (which is refereed as data size in the manuscript). Please revise to systematic. | Thanks for pointed out this, we have changed in revised manuscript |
|  | 9. Please clarify self-made form | we have replaced the present from the following statement  ***Self-designed structured pre-tested questionnaires***. |
|  | 10. How was the questionnaire developed and validated? | The questionnaire was developed with the help of psychologist and the statement also added regarding this in our revised manuscript |
|  | 11. Please mention about calibration of instruments as well. | The questionnaire was developed with the help of psychologist and tested into 15 adolescents for validity. the statement also added regarding this in our revised manuscript |
|  | 12. Findings need to be rewritten and presented in a comprehensive manner. | We have tried to revised and make it more comprehensive and related to the objectives |
|  | 13. Please rewrite conclusion and recommendations section. | We have tried to revised and make it more comprehensive and related to the objectives |
|  | 14. The authors need to edit the entire manuscript for language and grammar. | We have referred English grammar rectification tool to omit our mistakes |

Thanks Editor, Reviewer 1 and Reviewer 2 for your kind suggestions
