# Supplementary material for: Anxiety levels among school-going adolescents in peri-urban areas of Karachi, Pakistan
Source: PLoS One. 2023 Oct 19;18(10):e0289967. doi: 10.1371/journal.pone.0289967 (PMC10586665; doi:10.1371/journal.pone.0289967)
Supplement: Attachment — Submitted filename: plos one review.docx [file pone.0289967.s003.docx]

Review:

Anxiety levels among school-going adolescents in peri-urban areas of Karachi, Pakistan

Overall comments:

1. Please re-write the conclusion in the abstract section.
2. Please edit the manuscript for language and grammar.
3. Please rewrite the ‘what this study adds’ section in the revised manuscript.

Introduction:

1. Please consider embedding rationale and objectives into the introduction section.
2. The objectives could be rewritten comprehensively.

Methods:

1. ‘A systemic random sample’- please revise it to systematic and mention how systematic sampling was undertaken in the text.
2. ‘we assumed the majority as 50% through a literature search,’: please check and cite references for the literature review undertaken
3. Under sample size calculation: The text mentions ‘Patient sample size’- who are the patients here?
4. Pilot study was undertaken among 15 students on what basis? At least 10-20% of the sample should have been considered.

Discussion:

1. Authors may consider adding implications of the study

Strengths:

1. ‘By doing this kind of study, we can solve different mental issues in adolescents of a highly vulnerable state because Patients with mild cognitive Anxiety might be particularly vulnerable and lead to anxiety disorder’- how ??
