# Supplementary material for: Anxiety levels among school-going adolescents in peri-urban areas of Karachi, Pakistan
Source: PLoS One. 2023 Oct 19;18(10):e0289967. doi: 10.1371/journal.pone.0289967 (PMC10586665; doi:10.1371/journal.pone.0289967)
Supplement: Attachment — Submitted filename: Response to Reviewers.docx [file pone.0289967.s004.docx]

Reviewer 2 Query

| **Reviewer Query** | **Response from the authors** |
| --- | --- |
| 1.Please re-write the conclusion in the abstract section. | Thank you for highlighting this, we have revised the conclusion please see pg 2 |
| 2.Please edit the manuscript for language and grammar. | Noted!, We have considered the English editing service to omit the grammatical and language errors |
| 3.Please rewrite the ‘what this study adds’ section in the revised manuscript. Introduction: | Thank you for highlighting this, we have revised the conclusion please see pg 3 |
| 1.Please consider embedding rationale and objectives into the introduction section. | Thank you for highlighting this, we have revised the conclusion please see pg 3-4 |
| 2.The objectives could be rewritten comprehensively. | Thank you for highlighting this, we have revised the conclusion please see pg 4 |
| Methods: 1.‘A systemic random sample’- please revise it to systematic and mention how systematic sampling was undertaken in the text. | Thank you for highlighting this, we have revised the conclusion please see pg 4 |
| 2.‘we assumed the majority as 50% through a literature search,’: please check and cite references for the literature review undertaken | Thank you for highlighting this, we have revised the conclusion please see pg 4 |
| 3.Under sample size calculation: The text mentions ‘Patient sample size’- who are the patients here? | Apology for the typo error we have rectified and replaced by students |
| 4.Pilot study was undertaken among 15 students on what basis? At least 10-20% of the sample should have been considered. | Apology for the typo error! We have added 15% |
| Discussion: 1.Authors may consider adding implications of the study | Thank you we have tried to add some implications for this study |
| Strengths: 1.‘By doing this kind of study, we can solve different mental issues in adolescents of a highly vulnerable state because Patients with mild cognitive Anxiety might be particularly vulnerable and lead to anxiety disorder’- how ?? | Due to screening the anxiety level in students we can able to know if pupil have anxiety or not. After screening we can do further measures to prevent this state after screening and highlighted it to their parents so that further treatment can be done. |

Reviewer 3

| **Reviewer Query** | **Response from the authors** |
| --- | --- |
| The manuscript is a eloquent read. The authors have addressed all the previous comments which were asked by previous reviewers. i found grammatical and language mistakes and which needs to be corrected before publication. | Thank you for the feedback!  We have considered the English editing service to omit the grammatical and language errors |
