# Supplementary material for: Anxiety levels among school-going adolescents in peri-urban areas of Karachi, Pakistan
Source: PLoS One. 2023 Oct 19;18(10):e0289967. doi: 10.1371/journal.pone.0289967 (PMC10586665; doi:10.1371/journal.pone.0289967)
Supplement: Attachment — Submitted filename: Response to editor.docx [file pone.0289967.s005.docx]

| Clarity in object: The objective is stated but could be made clearer by specifically mentioning what the study aims to achieve, such as understanding the prevalence of anxiety, identifying risk factors, or assessing the impact of anxiety on adolescents' well-being. | Thank you for your guidance. We rectified this in our manuscript on page no. 02 |
| --- | --- |
| Methodology: Provide more details about the sampling method and how the schools were selected. It would be helpful to mention the total number of schools approached and the criteria used for inclusion in the study. | We have revised the methodology and add the sampling procedure to clear the details. Thank you! For pointing out this. |
| Missing Information: The abstract lacks information on the response rate or participation rate of the students in the study. Including this data would give readers an idea of the representativeness of the sample. | We have added the missing information in abstract now. Thank you! |
| Results: The findings mentioned in the abstract should be more specific. For example, provide the total number of participants who had moderate to severe anxiety symptoms, and clearly state the prevalence rates among males and females. | Thank you for pointing out this. We modified this in our manuscript on page no: 02 |
| Statistical Significance: If possible, mention whether the associations between variables and anxiety levels were statistically significant to add more credibility to the findings. | We have added the lines for associations in the abstract. Thank you! |
| Implications: The abstract could benefit from briefly discussing the potential implications of the study's findings. How could the results help in developing intervention programs or improving mental health services for adolescents in peri-urban areas? | Thank you for pointing out this. We modified this in our manuscript on page no: 02 |
| Conclusion: The conclusion is clear in summarizing the key findings, but it could be strengthened by providing a brief recommendation for future research or practical implications for addressing adolescent anxiety. | Thank you for your guidance. We modified this in our manuscript on page no: 02 |
| The introduction provides a clear overview of the importance of studying anxiety among adolescents and the lack of research in peri-urban areas of Karachi, Pakistan. | Thank you for your kind words and appreciation. |
| The introduction successfully highlights the global prevalence of anxiety disorders among adolescents, providing relevant statistics. | We are thankful to you for this appreciation |
| However, it would be helpful to include the specific sources for the cited statistics to enhance the credibility of the information. | Mis sana |
| There seems to be repetition of information regarding the GAD-7 scale and its psychometric qualities. Consider consolidating this information into a concise paragraph. | Thank you for your guidance. We rectified this in our manuscript on page no. 03 |
| The mention of the recent studies conducted in the USA and Europe using the GAD-7 scale strengthens the case for using this tool for anxiety screening. However, it might be beneficial to provide a brief summary of their key findings. | Thank you for highlighting this. We rectified this in our manuscript on page no. 03 |
| The rationale for the study is well-explained, focusing on the lack of mental health screening for school-going adolescents in peri-urban areas of Karachi, Pakistan, and the specific challenges they face. | Thank you for your feedback. |
| Consider including more specific information about the challenges faced by adolescents in peri-urban areas of Karachi, such as economic difficulties, lack of mental health resources, and the impact of the COVID-19 outbreak. | Thank you for highlighting this. We rectified this in our manuscript on page no. 03 |
| The objectives of the study are clearly stated, addressing the different factors that may contribute to anxiety levels among school-going adolescents. | Thanks for your feedback. |
| It would be beneficial to include a brief overview of the methodology, such as the sample size, age group, data collection methods, and statistical analysis techniques to be used. | Thank you for pointing out this! We have revised the methodology section please see page# 5 |
| Ensure that the use of references is consistent throughout the introduction. | Thank you for highlighting this. We rectified that. Please see page no: 10. All the references numbers are in sequence now |
| Overall, the introduction provides a solid foundation for the study, highlighting the significance of addressing anxiety among school-going adolescents in peri-urban areas. By addressing the points mentioned above, the introduction can be further improved for a more comprehensive and compelling overview of the research | Thank you for your feedback. We addressed the above points which you mentioned above. |
| Methodology. The study site and sample selection process are well-explained, providing relevant information about the three selected slum areas and the rationale behind their selection | Thank you for your feedback |
| The study's ethics approval and consent process are clearly described, ensuring that participants' identities remain confidential. | Thank you for your feedback |
| The justification for the sample size calculation is provided, considering a prevalence rate of 50% and a 20% no response rate. However, it would be helpful to include a brief explanation of the assumptions made in choosing these values. | There is no such study in Pakistan especially in resource-poor settings. We have assumed the over all anxiety in 50% prevalence so that It would be easy for us to summarize the results with no bias |
| The inclusion and exclusion criteria are clearly stated, defining the age range, socio-economic status, and consent requirements for participation. | Thanks for your kind feedback |
| The description of the data collection tools, including the demographic form and the GAD-7 scale, is well-presented, providing clarity on what information is being collected. | Thank you for your feedback |
| The validation process of the self-designed structured questionnaire through a pilot survey is a good approach to ensure the validity of the instrument. | Thank you for your appreciation |
| The data collection process is well-outlined, mentioning the schools' selection, translation of the questionnaire, and the involvement of researchers in administering the survey. | Thank you for your feedback |
| The anthropometric measurements section provides details on how blood pressure measurements were taken and validated, which adds to the study's credibility. | Thank you for your feedback |
| The statistical analysis plan is comprehensive, covering data review, descriptive statistics, bivariate analysis, and multivariate logistics regression to identify significant predictors of anxiety levels. | Thank you for your feedback |
| It would be helpful to include a brief mention of the data analysis software used (SPSS version 24) and any statistical tests that will be employed. | Thank you! It has been mentioned in the first two lines under the heading of statistical analysis. Please refer page # 6 |
| Consider adding information about any measures taken to ensure data quality and minimize biases during data collection. | Thank you for pointing out this, we have add after result section in page # 7 |
| Ensure that all figures and tables mentioned in the text are included and appropriately labeled. | Thank you for highlighting this. We corrected this. Please see page no: 14,15,16,17 |
| Overall, the methodology section is well-structured and provides sufficient details for understanding the study's design and data collection process. By addressing the points mentioned above, the methodology section can be further enhanced for a comprehensive and robust study. | Thank you for your feedback. We rectified all the above-mentioned points. |
| Results: The introduction of the results section provides a clear summary of the participant recruitment process, with 700 participants approached and 544 participants included for final analysis. | Thank you for your feedback. |
| Table 1 is mentioned, which shows socio-demographic analysis. However, it would be more informative if the key findings from Table 1 are briefly described in the text, highlighting the relevant demographic characteristics of the participants. | Thank you for highlighting this point. We rectified this. Please check page no: 6 |
| The use of odds ratios (OR) and confidence intervals (CI) to demonstrate the association between anxiety levels and various factors is appropriate. However, it is essential to interpret these results carefully, considering the wide confidence intervals and the potential for non-significant associations. | We have modified the OR in the result section. Please see page # 6Thank you! |
| The results describe the association between anxiety levels and factors such as gender, food intake, presence of a smoker in the family, and parenting style. However, it would be more informative to present the actual values of OR and CI, not just stating whether they are significant or not. | Thank you for pointing out this. We rectified this in our manuscript. Please see page # 6 |
| Provide more clarity on the interpretation of the OR values. For example, an OR of 1.010 suggests a very minimal difference, so it is important to specify the practical significance of such results. | We have modified the result section and made it more sound. Please refer page 6. Thank you! |
| When mentioning percentages, ensure that the total sample size for each gender is specified for better context and interpretation. | We have added the total sample size of gender. Thank you! |
| It would be helpful to include additional statistical measures, such as p-values, to determine the significance of the observed associations. | We have added the associations with p-value. Thank you! |
| The description of Table 4, showing anthropometric measurements, is brief and lacking in interpretation. Consider discussing any noteworthy findings or patterns in these measurements among adolescents | Thank you for the suggestion! We have discussed in detail in page 9 last paragraph of discussion section. |
| Overall, the results section is informative, but additional details and interpretation of the findings would enhance its clarity and significance. | T Thank you for your valuable comments, we have now modified the results section as per your instructions. |
| Consider organizing the results section with subheadings to make it more structured and reader-friendly, especially when referring to different tables. | Thank you for highlighting this. We have added the headings as per your valuable instructions |
| Discussion: The discussion starts with a relevant and concise introduction that highlights the significance of mental health concerns among children and adolescents globally. | Thank you for your feedback. |
| The first point discusses the higher prevalence of anxiety in females, which is supported by previous studies. However, it would be beneficial to elaborate on potential reasons for this gender difference in anxiety, such as hormonal factors, societal expectations, or coping mechanisms. | Thank you for highlighting this. We rectified this in our manuscript. Please see discussion section at page no: 7 |
| The discussion on parenting style and its influence on anxiety is well-presented, citing studies from different countries. To strengthen this point, consider discussing specific parenting practices associated with higher anxiety levels and potential interventions to promote healthier parenting styles. | Thank you for your response. We have modified this. Please check page no: 7 |
| When mentioning previous studies, ensure accurate referencing by providing complete citation details, including authors, year, and journal or publication. | Thank you for pointing out this. We have rectified this in our manuscript. |
| The discussion on sedentary lifestyles, gaming, and transportation as factors influencing anxiety is informative. Consider discussing the underlying mechanisms behind these associations, such as increased screen time affecting sleep patterns or reduced physical activity affecting stress management. | Thank you for pointing out this. We have rectified this in our manuscript. |
| To strengthen the discussion on adolescents from smoking families, provide further insights into the potential impact of secondhand smoke exposure on anxiety levels in adolescents. Discuss possible interventions to support families in reducing smoking and promoting a healthier environment for adolescents. | Thank you for pointing out this. We have rectified this in our manuscript. See page no: 8 |
| The impact of food intake on anxiety is well-explained, supported by relevant studies. To enhance this point, discuss the role of specific nutrients and dietary patterns in influencing mood and anxiety in adolescents. | Thank you for pointing out this. We have rectified this in our manuscript. See page no: 8 |
| The discussion on tuition and academic stress is valuable. Expand on potential reasons why tuition attendance may be associated with higher anxiety levels. Discuss strategies to promote academic support while minimizing stress among students. | Thank you for pointing out this. We have rectified this in our manuscript. See page no: 8 |
| The comparison of BMI and anxiety levels is relevant. However, provide a more nuanced analysis of the relationship, considering potential confounding factors, such as age, gender, and lifestyle habits. Discuss how BMI changes over time may influence anxiety outcomes. | This we discussed in little detailed in discussion part last paragraph at page # 9 |
| Consider adding a final section summarizing the key findings and implications of the study. Discuss how the results contribute to the existing body of knowledge on adolescent anxiety and suggest potential interventions or areas for further research. |  |
| Overall, the discussion is well-structured, and the inclusion of supporting studies adds credibility to the presented findings. To enhance the section, provide more in-depth analysis and interpretation of the results, while also highlighting potential avenues for future research and practical implications for mental health interventions. | Thank you for highlighting this point. We have made changes in discussion part. Please check page no: 8 |
